# Inhibitory effect of *Xenorhabdus* *nematophila* TB on plant pathogens *Phytophthora capsici* and *Botrytis cinerea in vitro* and *in planta*

**DOI:** 10.1038/srep04300

**Published:** 2014-03-06

**Authors:** Xiangling Fang, Manrang Zhang, Qian Tang, Yonghong Wang, Xing Zhang

**Affiliations:** 1 Research and Development Center of Biorational Pesticides, Key Laboratory of Plant Protection Resources and Pest Management of Ministry of Education, Northwest A&F University, 22 Xinong Road, Yangling, Shaanxi 712100, China; 2 College of Horticulture, Northwest A&F University, 3 Taicheng Road, Yangling, Shaanxi 712100, China; 3 Shaanxi Research Center of Biopesticide Engineering and Technology, Northwest A&F University, 22 Xinong Road, Yangling, Shaanxi 712100, China; 4 These authors contributed equally to this work.

## Abstract

Entomopathogenic bacteria *Xenorhabdus* spp. produce secondary metabolites with potential antimicrobial activity for use in agricultural productions. This study evaluated the inhibitory effect of *X. nematophila* TB culture on plant pathogens *Botrytis cinerea* and *Phytophthora capsici*. The cell-free filtrate of TB culture showed strong inhibitory effects (>90%) on mycelial growth of both pathogens. The methanol-extracted bioactive compounds (methanol extract) of TB culture also had strong inhibitory effects on mycelial growth and spore germinations of both pathogens. The methanol extract (1000 μg/mL) and cell-free filtrate both showed strong therapeutic and protective effects (>70%) on grey mold both in detached tomato fruits and plants, and leaf scorch in pepper plants. This study demonstrates *X. nematophila* TB produces antimicrobial metabolites of strong activity on plant pathogens, with great potential for controlling tomato grey mold and pepper leaf scorch and being used in integrated disease control to reduce chemical application.

Plant diseases are a serious threat to global food production and security. Fungal and oomycete diseases are major concerns for the commercial production of vegetables, fruits and crops in china. The most common fungal and oomycete pathogens belong to the genera *Alternaria*, *Botrytis*, *Cochliobolus*, *Fusarium*, *Geotrichum*, *Penicillium*, *Sclerotina* and *Phytophthora*, of which *Botrytis cinerea* and *Phytophthora capsici* are two of the most economically important pathogens on vegetables responsible for serious economic losses^1^,^2^,^3^. These pathogens are mainly controlled by chemical fungicides that most are highly toxic and non-biodegradable. The heavy and widespread application of fungicides has not only resulted in the development of plant resistance to these chemicals, but also is a major source of environmental contamination and ecosystem damage^4^,^5^,^6^. It is necessary to develop alternative control strategies or new agrochemicals to control plant diseases in an effective and sustainable way.

*Xenorhabdus* spp., which belong to a unique genus of bacteria, are symbiotically associated with entomopathogenic nematodes *Steinernema* spp., and are carried in the intestine of the infective juvenile stage of nematodes. The infective juvenile penetrates an insect host and releases the bacteria into the insect hemocoel. The bacteria multiply rapidly and produce various metabolites that can overcome the insect immune system^7^, kill the insect and inhibit the growth of various fungal and bacterial competitors^8^,^9^,^10^. Such bacterial symbionts can prevent putrefaction of the insect cadaver and establish conditions favor the development of both the nematode and bacterial symbionts^11^.

Compounds with antimicrobial activity produced by *Xenorhabdus* spp. have been isolated and identified, including nonproteinaceous compounds (e.g., indoles, xenorhabdins, xenocoumacin, nematophin, benzylineacetone, xenortides, xenematide and cyclolipopeptide)^12^,^13^,^14^,^15^,^16^,^17^,^18^ and proteinaceous compounds (e.g., xenorhabdincin and chitinases)^19^,^20^,^21^. The nonproteinaceous groups are highly active on Gram-positive bacteria but less active on Gram-negative bacteria^16^,^17^,^19^,^22^, and some of them are active also on fungi^12^,^15^,^17^,^22^. The proteinaceous xenorhabdincin is selectively active on bacteria^21^ and also active against fungi^20^. Among *Xenorhabdus* spp., *X. nematophila* produce nematophin and xenocoumacin, which can inhibit the growth of *B. cinerea* and *P. infestans*^15^. Fourteen gene clusters involved in the biosynthesis of xenocoumacin have been identified by molecular genetic analysis^23^. However, the action modes of these antimicrobial compounds on fungal and oomycete pathogens are still unknown.

The inhibitory effect of *Xenorhabdus* spp. culture on some fungal and oomycete pathogens has been determined^9^,^22^,^24^,^25^,^26^,^27^,^28^,^29^. However, there are no studies on the potential inhibitory effect of *X. nematophila* culture systematically from *in vitro* to *in planta*. This study determined the inhibitory effect of *X. nematophila* TB culture toward a wide range of plant pathogenic fungal and oomycete pathogens *in vitro*, with special focus on the inhibitory effect of *X. nematophila* TB culture on *B. cinerea* causing grey mold in tomato plants and *P. capsici* causing leaf scorch in pepper plants.

## Results

### *In vitro* effect of cell-free filtrate on mycelial growth of different pathogens

**Figure 1:**
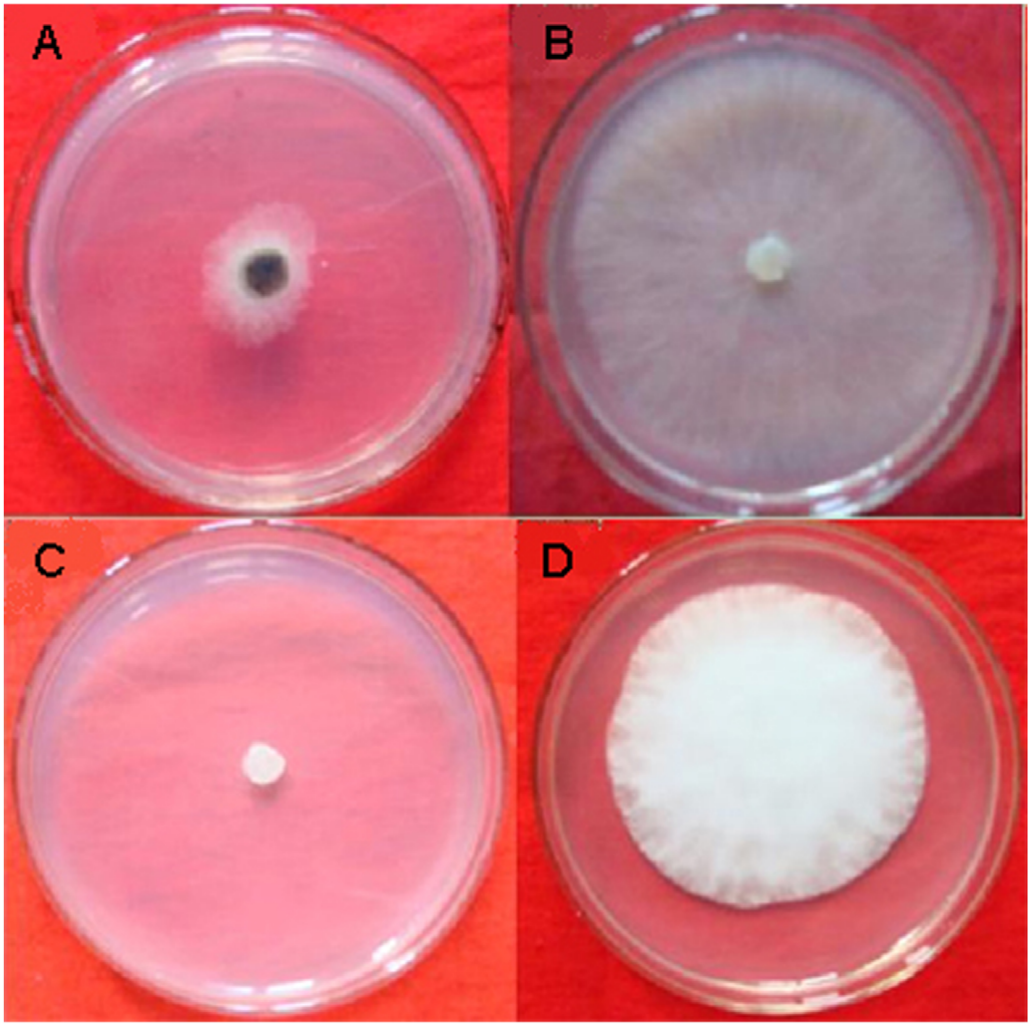
Effect of the cell-free filtrate of *Xenorhabdus nematophila* TB culture on mycelial growth of *Botrytis cinerea* and *Phytophthora capsici*. (A) *B. cinerea* growing on potato dextrose agar (PDA) mixed with cell-free filtrate of *X. nematophila* TB culture at 100 mL/L. (B) *B. cinerea* growing on PDA. (C) *P. capsici* growing on PDA mixed with cell-free filtrate of *X. nematophila* TB culture at 100 mL/L. (D) *P. capsici* growing on PDA.

The cell-free filtrate of *X. nematophila* TB culture showed a wide range of inhibitory effect from 15% to 100% on the mycelial growth of all the fungal and oomycete pathogens tested (Table 1). In particular, the cell-free filtrate of *X. nematophila* TB culture exhibited high inhibitory effect (>90%) on *Botrytis cinerea, Phytophthora capsici, Alternaria solani, Bipolaria maydis, Bipolaris sorokinian, Dothiorella gregaria, Exserohilum turcicum, Physalospora piricola, Rhizoctonia cerealis* and *Sclerotinia sclerotiorum*. Among these, *B. cinerea* and *P. capsici* were selected as the pathogens for subsequent studies (Fig. 1).

### *In vitro* effect of methanol extract on mycelial growth of *B. cinerea* and *P. capsici*

**Figure 2:**
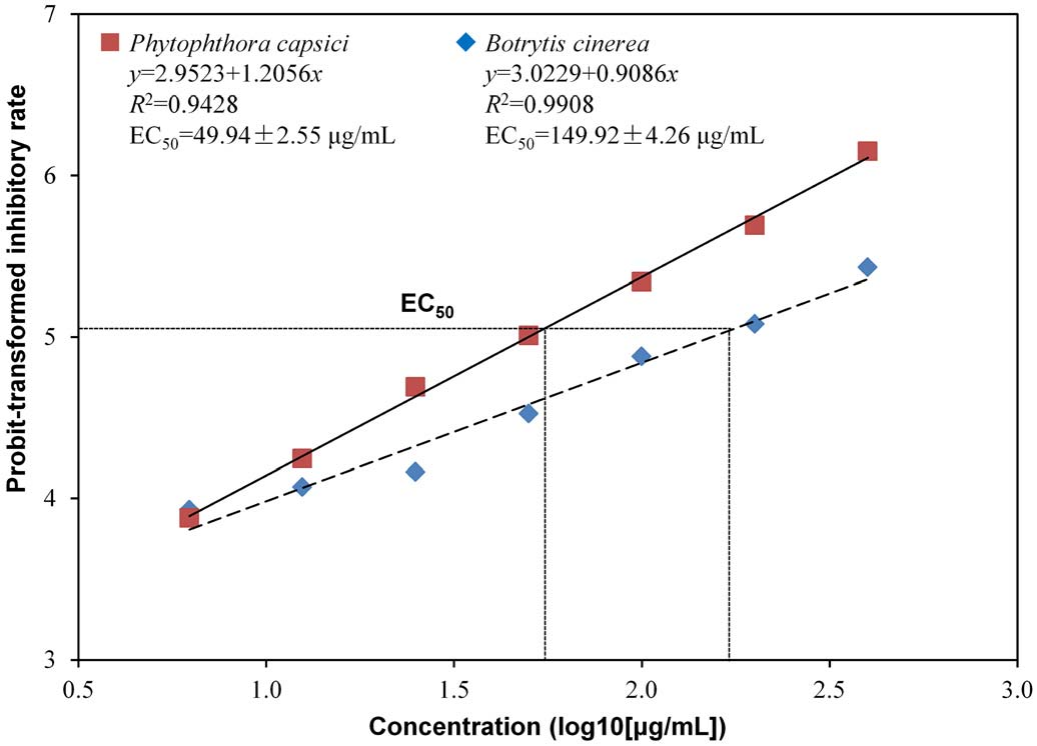
Effect of methanol extract of the cell-free filtrate of *Xenorhabdus nematophila* TB culture on mycelial growth of *Botrytis cinerea* and *Phytophthora capsici*. EC_50_, 50% effective concentrations.

The concentrations of methanol extract from 6.25 to 400 μg/mL all showed inhibitory effect on the mycelial growth of *B. cinerea* and *P. capsici*, and there was a liner relationship between log10-transformed concentration of methanol extract and probit-transformed inhibitory rate on the mycelial growth of *B. cinerea* (*y* = 3.0229 + 0.9086*x*, *R^2^* = 0.9908) and *P. capsici* (*y* = 2.9523 + 1.2056*x*, *R^2^* = 0.9428) (Fig. 2). The methanol extract showed stronger inhibitory effect on *P. capsici* than *B. cinerea* when its concentration was higher than 12.5 μg/mL where methanol extract at 1000 μg/mL (relative to 78 mL cell-free filtrate) showed an inhibitory rate of 87.5% (probit = 6.15) and 66.6% (probit = 5.43) on the mycelial growth of *P. capsici* and *B. cinerea*, respectively. The EC_50_ of methanol extract on *P. capsici* and *B. cinerea* was 49.94 and 149.92 μg/mL, respectively.

### *In vitro* effect of methanol extract on spore germination of *B. cinerea* and *P. capsici*

**Figure 3:**
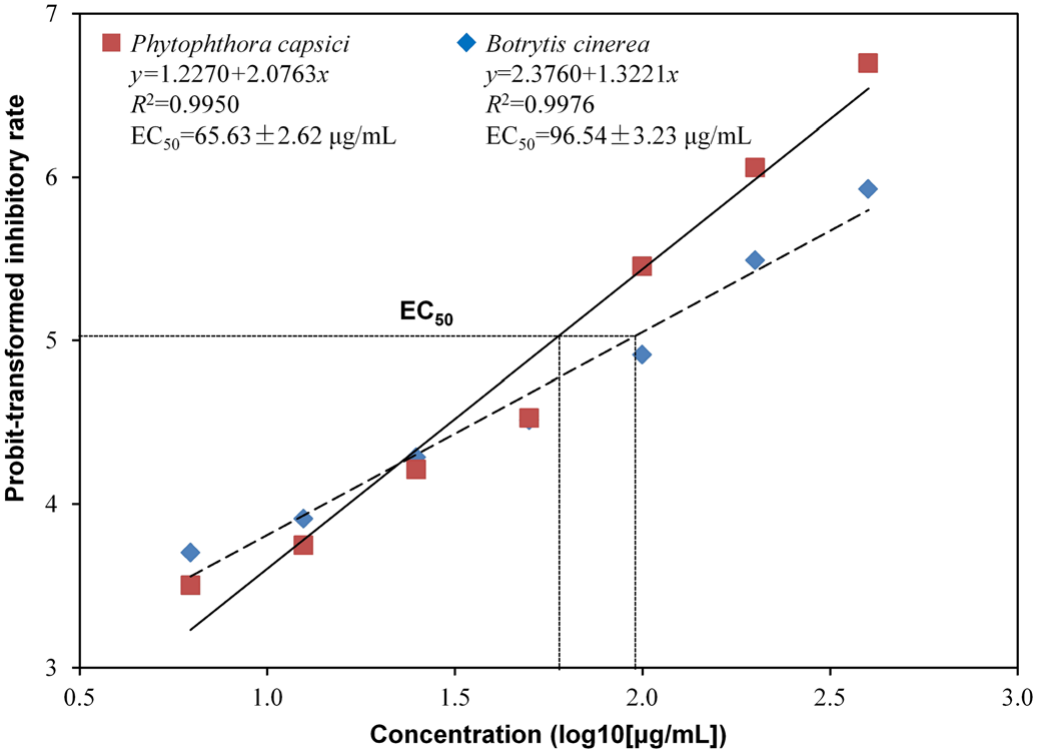
Effect of methanol extract of the cell-free filtrate of *Xenorhabdus nematophila* TB culture on spore germination of *Botrytis cinerea* and *Phytophthora capsici*. EC_50_, 50% effective concentrations.

The concentrations of methanol extract from 6.25 to 400 μg/mL all showed inhibitory effect on the spore germination of *B. cinerea* and *P. capsici*, and there was a liner relationship between log10-transformed concentration of methanol extract and probit-transformed inhibitory rate on the spore germination of *B. cinerea* (*y* = 2.3760 + 1.3221*x*, *R^2^* = 0.9976) and *P. capsici* (*y* = 1.2270 + 2.0763*x*, *R^2^* = 0.9950) (Fig. 3). The methanol extract showed stronger inhibitory effect on *P. capsici* than *B. cinerea* when its concentration was higher than 25 μg/mL where methanol extract at 1000 μg/mL showed an inhibitory rate of 95.5% (probit = 6.70) and 82.3% (probit = 5.93) on the spore germination of *P. capsici* and *B. cinerea*, respectively. The EC_50_ of methanol extract on *P. capsici* and *B. cinerea* was 65.63 and 96.54 μg/mL, respectively.

### Effect of methanol extract and cell-free filtrate on detached tomato fruits infected with *B. cinerea*

**Figure 4:**
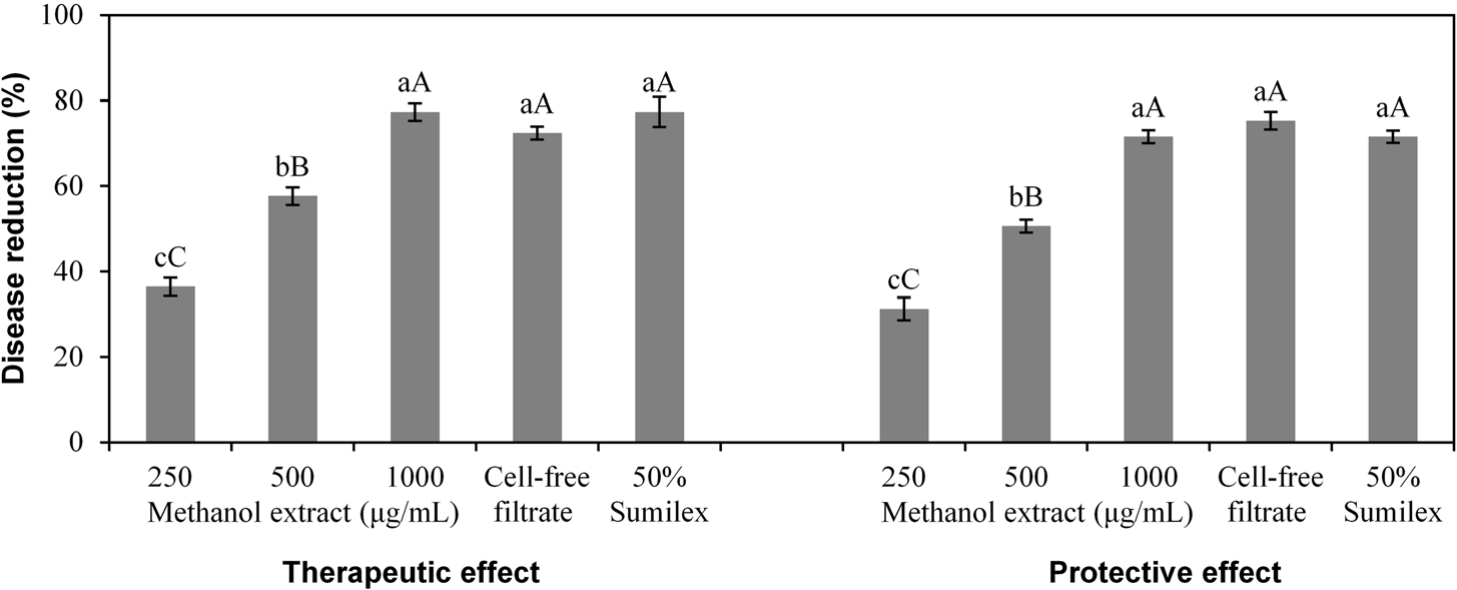
Effect of *Xenorhabdus* *nematophila* TB culture on grey mold of detached tomato fruits caused by *Botrytis cinerea*. For both therapeutic and protective effect, the treatments are methanol-extracted bioactive compounds (methanol extract) at 250, 500 and 1000 μg/mL, cell-free filtrate, and chemical control (50% Sumilex, 1000×). Data are presented as Mean ± SE (n = 6). Different lower case letters above the bars indicate significant differences at *P* = 0.05. Different capital letters above the bars indicate significant differences at *P* = 0.01.

**Figure 5:**
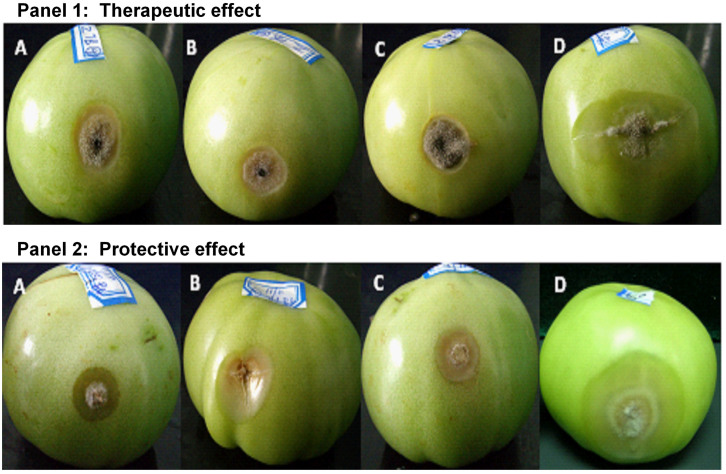
Detached tomato fruits inoculated with *Botrytis cinerea*. Panel 1 (therapeutic effect): tomato fruits inoculated with agar discs from the edges of 3 to 5-day-old *B. cinerea* colony growing on PDA 24 h prior to spray with (A) methanol-extracted bioactive compounds at 1000 μg/mL, (B) cell-free filtrate of *X. nematophila* TB culture, (C) chemical control (50% Sumilex, 1000×) and (D) water control. Panel 2 (protective effect): tomato fruits inoculated with agar discs from the edges of 3 to 5-day-old *B. cinerea* colony growing on PDA 24 h after spraying with (A) methanol-extracted bioactive compounds at 1000 μg/mL, (B) cell-free filtrate of *X. nematophila* TB culture, (C) chemical control (50% Sumilex, 1000×) and (D) water control.

There was a significant effect (*P* < 0.001) of treatments (viz. methanol extract at 250, 500 and 1000 μg/mL, cell-free filtrate and chemical control) on detached tomato fruits infected with *B. cinerea*, and at each treatment, there was no significant difference (*P* > 0.05) between therapeutic effect and protective effect (Fig. 4). The methanol extract at 1000 μg/mL exhibited 77.35% therapeutic effect and 71.57% protective effect on detached tomato fruits infected with *B. cinerea*, which were similar to the therapeutic effect and protective effect of the cell-free filtrate and the chemical control (50% Sumilex, 1000×). For both the therapeutic effect and protective effect treatment, tomato fruits sprayed with the methanol extract (1000 μg/mL), the cell-free filtrate and 50% Sumilex (1000×) showed only small legions while fruits sprayed with water showed large lesions (Fig. 5).

### Effect of methanol extract and cell-free filtrate on tomato plants infected with *B. cinerea* and pepper plants infected with *P. capsici*

**Figure 6:**
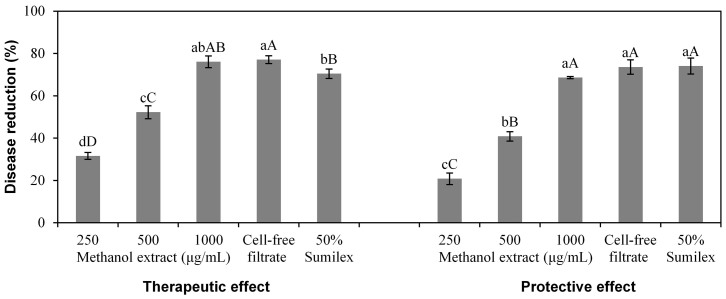
Effect of *Xenorhabdus* *nematophila* TB culture on grey mold in tomato plants caused by *Botrytis cinerea*. For both therapeutic and protective effect, the treatments are methanol-extracted bioactive compounds (methanol extract) at 250, 500 and 1000 μg/mL, cell-free filtrate, and chemical control (50% Sumilex, 1000×). Data are presented as Mean ± SE (n = 6). Different lower case letters above the bars indicate significant differences at *P* = 0.05. Different capital letters above the bars indicate significant differences at *P* = 0.01.

There was a significant effect (*P* < 0.001) of treatments (viz. methanol extract at 250, 500 and 1000 μg/mL, cell-free filtrate and chemical control) on tomato plants infected with *B. cinerea*, and there was no significant difference (*P* > 0.05) between therapeutic effect and protective effect at each concentration of the methanol extract and the cell-free filtrate (Fig. 6). Both the therapeutic effect and protective effect of the methanol extract at 1000 μg/mL and the cell-free filtrate were higher than 70%, which were similar to the therapeutic effect and protective effect of the chemical control (50% Sumilex, 1000×).

**Figure 7:**
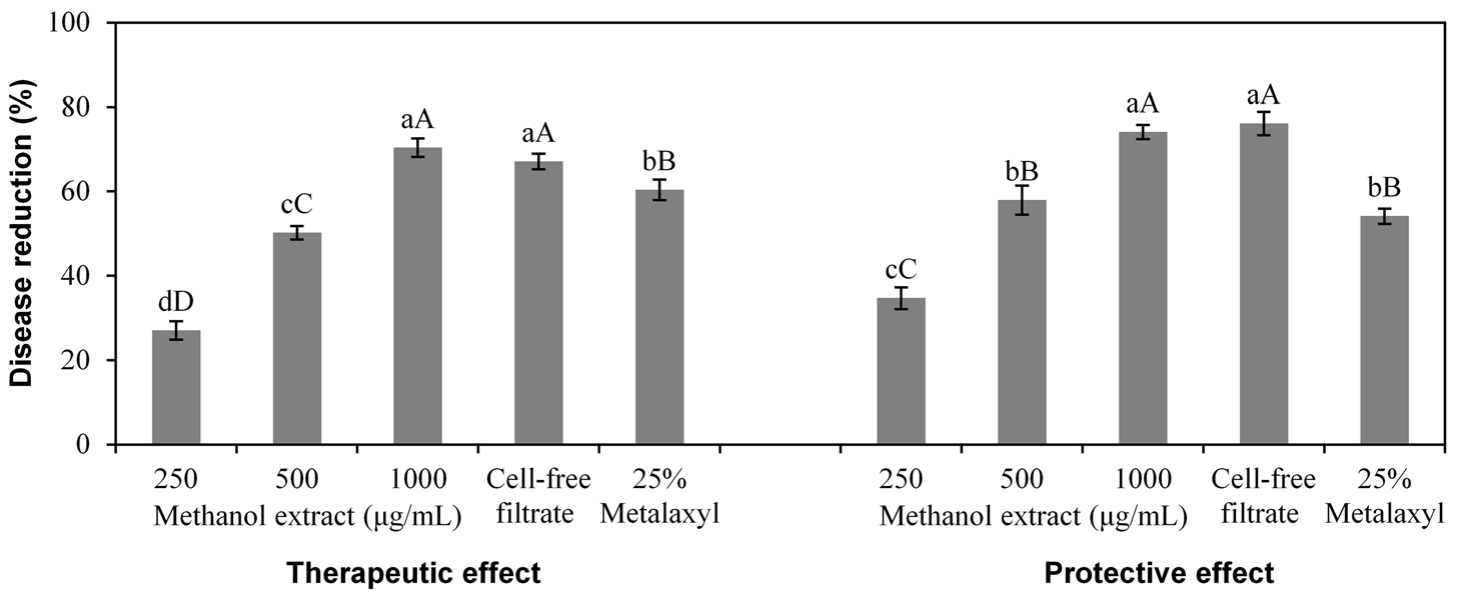
Effect of *Xenorhabdus* *nematophila* TB culture on leaf scorch in pepper plants caused by *Phytophthora capsici*. For both therapeutic and protective effect, the treatments are methanol-extracted bioactive compounds (methanol extract) at 250, 500 and 1000 μg/mL, cell-free filtrate, and chemical control (25% Metalaxyl, 500×). Data are presented as Mean ± SE (n = 6). Different lower case letters above the bars indicate significant differences at *P* = 0.05. Different capital letters above the bars indicate significant differences at *P* = 0.01.

There was also a significant effect (*P* < 0.001) of treatments (viz. methanol extract at 250, 500 and 1000 μg/mL, cell-free filtrate and chemical control) on pepper plants infected with *P. capsici*, and there was no significant difference (*P* > 0.05) between the therapeutic effect and protective effect at each concentration of the methanol extract and the cell-free filtrate (Fig. 7). At 1000 μg/mL, both the therapeutic effect and protective effect of the methanol extract and the cell-free filtrate were higher than 70%, which were stronger than the chemical control (25% Metalaxyl, 500×) in both therapeutic effect and protective effect that were lower than 61%.

## Discussion

The cell-free filtrate of *X. nematophila* TB culture exhibited a wide range of inhibitory effect on plant pathogenic fungi and oomycetes *in vitro*. Previous studies have reported the variation in antimicrobial activities of different *Xenorhabdus* spp. strains to fungal pathogens^9^,^18^,^24^,^27^,^35^,^36^,^37^,^38^,^39^. *B. cinerea* and *P. capsici* are the frequently reported pathogens associated with tomato grey mold and pepper leaf scorch, respectively, which result in marked agricultural economic losses annually in China^40^,^41^. This study found that the cell-free filtrate of *X. nematophila* TB culture showed strong inhibitory effect on the growth of *B. cinerea* and *P. capsici*. And thus, we focused on these two pathogens for both *in vitro* and *in planta* studies.

The methanol extract of *X. nematophila* TB culture not only inhibited the mycelial growth but also inhibited the spore germination of *B. cinerea* and *P. capsici*. The inhibitory effect of the methanol extract on the mycelial growth of *P. capsici* is stronger than *B. cinerea*, with an EC_50_ of 49.94 and 149.92 μg/mL, respectively. Similarly, the inhibitory effect of the methanol extract on the spore germination of *P. capsici* is stronger than *B. cinerea*, with an EC_50_ of 65.63 and 96.54 μg/mL, respectively. A previous study found that the inhibitory effect of the methanol extract of *X. bovienii* YL002 culture on both the mycelial growth and spore germination of *P. capsici* was weaker than on *B. cinerea*^33^. This difference may be due to different antimicrobial compounds produced by the two species of *Xenorhabdus* (*X. nematophila* and *X. bovienii*). *X. bovienii* mainly produces indoles and dithiolopyrrolones that exhibited both antifungal and antibacterial activity, but the antifungal activity of indoles is stronger than the antibacterial activity^16^,^22^. It has been reported that *Phytophthora* is resistant to most common antifungal compounds, but are sensitive to antibacterial compounds^42^. *X. nematophila* produces antimicrobial peptides (xenocoumacins, xenortides, xenematide and cyclolipopeptide), nematophin and benzylineacetone. Xenocoumacins, the major antibiotics produced by *X. nematophila*, can inhibit the growth of *P. capsici* and *B. cinerea* and the sporangia production of *P. infestans*^16^,^29^. Xenematide only exhibited a noticeable antibacterial effect^14^. Cyclolipopeptide exhibited antifungal activity on *B. cinerea*, *Phytophthora myc* and *P. oryzae*^12^. Nematophin showed significant inhibitory effect on *B. cinerea* and *P. infestans*^15^. Benzylineacetone is active against Gram-negative bacteria^13^. Thus, the methanol extract of *X. nematophila* TB culture showed stronger inhibitory effect on *P. capsici* than on *B. cinerea* while the methanol extract of *X. bovienii* YL002 culture showed stronger inhibitory effect on *B. cinerea* than on *P. capsici*.

The active compounds produced by *Xenorhabdus* spp. may act on *P. capsici* and *B. cinerea* by different cellular action mechanisms. *P. capsici* is an oomycete and differs from the fungi in cell wall composition that contains or produce sterols^43^. The active compounds xenocoumacins can act on *P. capsici* by reducing the activity of transporters involved in transport systems of *P. capsici* cell membranes^44^. *B. cinerea* is an ascomycete pathogen and spores of *B. cinerea* are considered to be the main source of dispersal of inoculum, and their germination and adhesion on plant surfaces represent crucial steps preceding host penetration and colonization^45^,^46^. The active compounds produced by *X. nematophlia* TB may affect the cellular process required for the germination of *B. cinerea* spores by multisite inhibitors or interfere with respiration, and thus *B. cinerea* cannot grow and reproduce normally to penetrate plant host. It has been found that *B. cinerea* mycelia treated with the active compounds from *X. nematophlia* YL001 culture showed morphological and structural alterations such as swollen beads, septa malformation, cell wall dissolution and cell liquid seepage^47^.

This study found that the *in vitro* and *in planta* effect of the methanol extract of *X. nematophila* TB culture on *B. cinerea* and *P. capsici* showed difference. The inhibitory effect of the methanol extract on both the mycelial growth and spore germination of *P. capsici* was stronger than on *B. cinerea in vitro*, but there was no obvious difference between its effect on tomato plants infected with *B. cinerea* and pepper plants infected with *P. capsici in planta*,. Traditionally, many studies on the antimicrobial activities of *Xenorhabdus* spp. are determined by *in vitro* assays that can give an indication of the inhibitory potency quickly, while there are few studies on plant pathogens *in planta*^48^. Since *in vitro* results do not always represent the antimicrobial activities of *Xenorhabdus* *in planta* situation, the potency determined in *in vitro* assays may be overestimated or underestimated compared with *in planta* situation, where pathgen infection is a well-regulated phenomenon that requires cross talk between host and pathogen through signals probably located on the external surfaces of cells^49^. The disturbance of cell membranes of pathogens by antimicrobial compounds apparently could lead to interference with such signals, which could eventually result in the failure of infection and development of symptoms on plants.

This study demonstrated that the application of methanol extract of the cell-free filtrate of *X. nematophila* TB culture could control tomato grey mold and pepper leaf scorch. The inhibitory effect of the methanol extract on the severity of grey mold and leaf scorch increased with the increasing concentration of methanol extract. Detached tomato fruits, tomato plants and pepper plants treated with the methanol extract 24 h prior to inoculation prevented the further establishment and expansion of the pathogen on the fruit or leaf surface. This suggests that the methanol extract can retain its antimicrobial activity for at least 24 h after being applied to fruits or leaves. Moreover, the methanol extract exhibited similar efficiency on both plant diseases tested in this study. Thus, the methanol extract of the cell-free filtrate of *X. nematophila* TB culture may be used to control plant diseases in agricultural production system. The methanol extract not only has strong activity on plant pathogens *in vitro* but also has strong control effect on plant diseases *in planta*. These justify the necessity of additional work to determine the effect of the methanol extract on tomato grey mold caused by *B. cinerea* and pepper leaf scorch caused by *P. capsici* in the field. Further work will also be conducted on using nonpolar solvents to extract active compounds and on identifying active compounds from *X. nematophila* TB culture to find alternate ways to reduce the application of chemical fungicides for sustainable agriculture production.

In summary, the cell-free filtrate and methanol extract of the cell-free filtrate of *X. nematophila* TB culture are of strong inhibitory effect on the plant pathogens *B. cinerea* and *P. capsici* both *in vitro* and *in planta*. *X. nematophila* TB culture has the great potential for controlling grey mold in tomato and leaf scorch in pepper and can be used in the integrated control of these pathogens with the objective of reducing the amount and number of chemical fungicide applications.

## Methods

### Bacterium strain and culture conditions

*X. nematophila* TB was isolated from its nematode symbiont *S.* *carpocapsa*e TB obtained from the soil collected from Taibai Mountain, Qinling, China^28^. This strain was firstly identified to be *X. nematophila* according to its morphological characteristics^28^, and confirmed to be *X. nematophila* by molecular identification conducted by PCR amplification and cloning of 16S rRNA gene, and the 16S rRNA gene sequence was then compared with other sequences in GenBank using the BLAST algorithm^30^. The 16S rRNA gene sequence of *X. nematophila* TB was deposited in GenBank (Accession number: EU124383).

*X. nematophila* occurs in two phases where phase I exhibits stronger antibiotic activity than Phase II^31^, and thus was used in this study. This strain is deposited in the Agricultural Culture Collection Institute, Northwest A&F University, China. Glycerinated stocks of this strain store at −70°C were used as starting material for subculture. To ensure the presence of phase I, this strain was subcultured onto plates with NBTA media [nutrient agar (NA, g/L) consisting of peptone 10, beef extract 3, NaCl 5 and agar 15 supplemented with 0.04% triphenyltetrazolium chloride (w/v) and 0.025% bromothymol blue (w/v)] and incubated at 28°C in darkness. The NBTA media can differentiate the phase I and phase II of this bacterium where phase I is of green colony while phase II is of red colony after 3 to 5 days' culture. Seed culture of *X. nematophila* TB was prepared by inoculating a loopful of phase I colony growing on NBTA plate into a 250 mL flask containing 50 mL fresh NB (NA without agar), which was adjusted to a final pH of 7.2, and then cultivated at 28°C in darkness on an Eberbach rotary shaker at 150 rpm for 18 to 24 h, during which time the optical density (600 nm) and pH readings were approximately 2.0 and 7.0, respectively.

### Cell-free filtrate and methanol extract of cell-free filtrate of *X. nematophila* TB culture

Batch cultures of *X. nematophila* TB were carried out in four 5 L fermenters (Eastbio, China). Each fermenter was equipped with one six-blade disk turbine impeller, pH probes (Mettler-Toledo GmbH, Switzerland), dissolved oxygen (DO) probes (Mettler-Toledo GmbH, Switzerland), a thermometer and foam. Seed culture (300 mL) was transferred into each fermenter containing 3.5 L autoclaved fermentation medium (g/L: glucose 6.13, peptone 21.29, MgSO_4_·7H_2_O 1.50, (NH_4_)_2_SO_4_ 2.46, KH_2_PO_4_ 0.86, K_2_HPO_4_ 1.11 and Na_2_SO_4_ 1.72). The pH of the medium was adjusted to 7.0 using 2.0 mol/L NaOH and 2.0 mol/L HCl. The fermenters were incubated at 28°C with the aeration rate of 2.5 L/min and the agitation speed of 300 rpm. After 72 h, the culture was transferred to centrifuge bottles, and centrifuged for 20 min (13200 g, 4°C) to get cell-free filtrate and stored at 4°C until required.

The methanol extract of the cell-free filtrate of *X. nematophila* TB culture was prepared based on methods described^24^,^32^,^33^. Briefly, D101 polymeric adsorbent resin (10 g, Bengbu Tianxing Ion-Resin Co. Ltd., China) were suspended in 100 mL sterile distilled water, then treated with 1% sterile HCl and 1% sterile NaOH with one hour each. After three washes with sterile distilled water, the pH was adjusted to 7.5 and kept at 4°C for 24 h. The cell-free filtrate was mixed with activated D101 polymeric adsorbent resin at 1:20 and incubated for 24 h. The resin slur was separated by a G3 glass filter, washed with distilled water and 25% methanol, and then placed on the top of the column filled with activated D101. After washing the column with distilled water, methanol was pumped onto the column at 500 mL/min and the eluate was collected in 200 mL aliquots. The methanol extract was dried at 40°C and stored at 4°C until required, and the average yield of the methanol extract of the cell-free filtrate was 12.8 g/L.

### Effect of cell-free filtrate on the mycelial growth of different pathogens

Twenty-six fungal and oomycete pathogens as listed in Table 1 were used in this test. These pathogens were obtained from the Agricultural Culture Collection Institute, Northwest A&F University, China, and were selected based on their importance associated with plant diseases in Shaanxi Province, China. These pathogens were subcultured onto fresh autoclaved potato dextrose agar (PDA) plates and maintained at 25°C in darkness. To determine the effect of cell-free filtrate of *X. nematophila* TB culture on these pathogens, cell-free filtrate was mixed with autoclaved PDA that had been cold down to about 70°C at 1:9, and then poured onto 9-cm Petri dish plates (10 mL mixture per plate). One mycelial disk (0.6 × 0.6 cm) from the edge of 3 to 5-day-old-colony of each pathogen growing on PDA was put onto the center of each plate. For each pathogen, there were three replicates (one plate per replicate), and the control plates for comparison were PDA only. The plates were maintained at 25°C in darkness. After 7 days, the colony diameter of each plate was measured, and the inhibitory rate was determined as described^33^. This experiment was repeated once under the same conditions.

### Effect of methanol extract on the mycelial growth of *B. cinerea* and *P. capsici*

A stock solution of the methanol extract (4000 μg/mL) of the cell-free filtrate of *X. nematophila* TB culture was prepared by dissolving the dried methanol extract in distilled water. Two-fold dilutions were made from the filter-sterilized stock solution. For each dilution, 1 mL was thoroughly mixed with 9 mL PDA and poured into a Petri dish plate. The final concentrations of the methanol extract in PDA were 6.25, 12.5, 25, 50, 100, 200 and 400 μg/mL. One mycelial disk (6-mm-diameter) from the edge of 3 to 5 day-old-colony of each pathogen growing on PDA was put onto the center of each plate. For each concentration, there were three replicates (one plate per replicate), and the control plates for comparison were PDA only. The plates were maintained at 25°C in darkness. The colony diameter of each plate was measured and the inhibitory rate for each concentration on each pathogen was determined after 7 days. This experiment was repeated once under the same conditions.

### Effect of methanol extract on the spore germination of *B. cinerea* and *P. capsici*

Spores of *B. cinerea* and *P. capsici* were collected from 5 to 7 day-old-colonies growing on PDA in darkness at 25°C by flooding each plate with 10 mL sterile distilled water containing 0.1% (v/v) tween 20 and rubbing the agar surface gently with a bent glass rod. The resulting spore suspension was filtered through four layers of Miracloth (Calbiochem, Merck Pty. Ltd., Australia). The spore concentrations were determined using a hematocytometer and adjusted to 1 × 10^8^ spores/mL in sterile distilled water for each pathogen. The methanol extract was dissolved in sterile distilled water, and diluted to obtain the concentrations of 12.5, 25, 50, 100, 200, 400 and 800 μg/mL. Methanol extract solution (200 mL) from each concentration were inoculated with spore suspension (200 mL) and then mixed thoroughly. The final concentrations of the methanol extract in the mixtures were 6.25, 12.5, 25, 50, 100, 200 and 400 μg/mL. The mixture (10 μL) from each concentration was placed on a glass slide. The control for comparison was spore suspension without methanol extract. There were three replicates (one slide per replicate) for each treatment. The slides were incubated in a moisture chamber at 25°C in darkness for 24 h. The number of spores germinated at each concentration was counted under the microscope. The percentage of spore germination was calculated, and the inhibitory rate was determined as described^33^. This experiment was repeated once under the same conditions.

### Effect of methanol extract and cell-free filtrate on detached tomato fruits infected with *B. cinerea*

Fresh green tomato fruits (cv. L402) with similar size (about 6-cm-diameter), picked from a glasshouse in Yangling (Shannxi Province, China), were used. Fruits were wounded on the middle sides using a sterilised needle (2-mm-diameter) before inoculation. To evaluate the therapeutic effect of methanol extract and cell-free filtrate of TB culture, mycelial agar discs (5-mm-diameter) from the edges of 3 to 5-day-old *B. cinerea* colony growing on PDA in darkness at 25°C were placed onto the pre-wounded sites of tomato fruits with the mycelial side facing the fruit (one mycelial disc per fruit). There were three replicates (eight fruits per replication) for each treatment. After inoculation, fruits were placed in plastic containers with moistened filter papers at the bottom to maintain high humidity. The containers were kept in a climate chamber at 25°C in darkness. After 24 h, the fruits were sprayed with 50 mL methanol extract at three concentrations (250, 500 and 1000 μg/mL) in water and the original cell-free filtrate. The controls were sprayed with water as negative control and with 50% Sumilex (1000×, Sumitomo Chemical Co. Ltd.) as positive control. To determine the protective effect, tomato fruits were sprayed with each solution prior to inoculation and kept under the same conditions as above. After 24 h, the fruits were inoculated with *B. cinerea* as above. The lesion diameter on each fruit was measured seven days post inoculation, and the inhibitory rate was determined as described^33^. This experiment was repeated once under the same conditions.

### Effect of methanol extract and cell-free filtrate on tomato plants infected with *B. cinerea* and pepper plants infected with *P. capsici*

Tomato seeds (cv. L402) were sown in plastic pots and kept in the controlled environmental room at 28/20°C (day/night) with about 70%-humidity and a 12 h-photoperiod (light intensity 350 μE m^−2^ s^−1^). Pepper Seeds (cv. Shijihong) were sown and kept in the controlled environmental room at 18/15°C (day/night) with about 70%-humidity and a 12 h-photoperiod (light intensity 250 μE m^−2^ s^−1^). Tomato plants with three true leaves and pepper plants with six true leaves were used. To determine the therapeutic effect, leaves of tomato plants and pepper plants were inoculated with spore suspensions (1 × 10^8^ spores/mL) of *B. cinerea* and *P. capsici*, respectively. Plants were covered with transparent polyethylene bags for 24 h to maintain high humidity, and plants were then sprayed with methanol extract at three concentrations (250, 500 and 1000 μg/mL) and the cell-free filtrate. For controls, plants were sprayed with water and commercial chemicals, respectively. Fifty percent Sumilex (1000×, Sumitomo Chemical Co. Ltd.) is commonly used for effective control of grey mold disease, while 25% Metalaxyl (500×, Sumitomo Chemical Co. Ltd.) is commonly used for effective control of leaf scorch disease on plants in China. There were three replicates (eight plants per replicate) for each treatment. To determine the protective effect, plants were sprayed with each solution as above prior to inoculation. After 24 h, plants were inoculated as above. After 15 days, the disease severity of plants were evaluated as described^33^,^34^. This experiment was repeated once under the same conditions.

### Data analyses

Data analyses were conducted using the SPSS statistical package (Version 11 for windows). Regression analyses were conducted to determine the relationship between the concentrations of methanol extract of the cell-free filtrate of *X. nematophila* TB culture and their inhibitory rate on the mycelial growth of *B. cinerea* and *P. capsici*, and also, the relationship between the concentrations of methanol extract and their inhibitory rate on spore germination of *B. cinerea* and *P. capsici*, and the associated regression equation, *R*^2^ and 50% effective concentration (EC_50_) of methanol extract were calculated. The concentrations of methanol extract were log10-tranformed and the inhibitory rates were probit-tranformed. Analyses of variance were conducted to determine the effects of treatments (viz. methanol extract at different concentrations, cell-free filtrate and chemical control) on the disease caused by *B. cinerea* in detached tomato fruits, the disease caused by *B. cinerea* in tomato plants and the disease caused by *P. capsici* in pepper plants. Subsequent multiple comparisons among treatments of each experiment were conducted based on Fisher's protected least significant differences at *P* = 0.05 and *P* = 0.01. Standard errors (SE) of means were also computed. For all analyses, data from the two repeat experiments (i.e., the original and one repeat experiment) of each study were not significantly different (*P* > 0.05); therefore, data from the two repeat experiments of each study were combined and analysed together.

## Author Contributions

Y.H.W. designed the experiments. X.L.F., M.R.Z. and Q.T. performed the experiments. X.L.F. and Y.H.W. interpreted the results. X.L.F., Y.H.W. and X.Z. wrote the paper.

## Figures and Tables

**Table 1:** The *in vitro* inhibitory effect of the cell-free filtrate of *Xenorhabdus nematophila* TB culture on the mycelial growth of different plant pathogens

Pathogens	Inhibitory rate (%)^a^	Pathogens	Inhibitory rate (%)^a^
*Alternaria alternate*	58.35 ± 1.72	*Fusarium graminearum*	37.13 ± 1.10
*Alternaria brassicae*	82.51 ± 0.68	*Fusarium oxysporum*	62.97 ± 1.12
*Alternaria brassicicola*	65.40 ± 1.25	*Gaeumannomyces graminis*	50.38 ± 0.87
*Alternaria solani*	90.73 ± 1.39	*Magnaporthe grisea*	77.2 ± 3.05
*Bipolaria maydis*	100.0 ± 000	*Penicillium digitatum*	83.40 ± 2.12
*Bipolaris sorokinian*	92.68 ± 3.07	*Physalospora piricola*	90.57 ± 1.25
*Botrytis cinerea*	90.39 ± 1.52	*Phytophthora capsici*	100.00 ± 0.00
*Clomerela cinyulate*	22.22 ± 0.55	*Rhizoctonia cerealis*	91.16 ± 1.05
*Colletotrichum lagenrium*	14.29 ± 0.38	*Rhizoctonia solani*	59.69 ± 0.72
*Didymella bryoniae*	45.49 ± 1.03	*Sclerotinia sclerotiorum*	97.35 ± 2.77
*Dothiorella gregaria*	93.02 ± 1.17	*Thanatephorus cucumeris*	87.33 ± 0.86
*Exserohilum turcicum*	91.54 ± 1.15	*Verticillium dahliae*	40.12 ± 0.15
*Fulvia fulva*	51.72 ± 0.85	*Verticillium dahliae* Kleb	37.31 ± 1.32

^a^The inhibitory rate of 100 mL/L cell-free filtrate of *X. nematophila* TB culture on the mycelial growth of the pathogens tested after 7 days. Data are presented as Mean ± SE (n = 6).
